# The correlation between ATP measurement and microbial contamination of inanimate surfaces

**DOI:** 10.1186/s13756-021-00981-0

**Published:** 2021-08-06

**Authors:** Andreas van Arkel, Ina Willemsen, Jan Kluytmans

**Affiliations:** 1 Department of Infection Control, Laboratory for Microbiology and Infection Control, Amphia Hospital, PO Box 90158, 4800 RK Breda, The Netherlands; 2 Laboratory for Microbiology and Infection Control, Elisabeth Tweesteden Hospital, Tilburg, The Netherlands; 3 Contrain – Infection Control Coach, Breda, The Netherlands; 4 Microvida Laboratory for Microbiology, Amphia Hospital, Breda, The Netherlands; 5 Julius Center of Health Sciences and Primary Care, UMC Utrecht, University Utrecht, Utrecht, The Netherlands

**Keywords:** ATP measurement, Surface contamination, Microbial contamination, Cleaning

## Abstract

**Background:**

The objective of this study was to determine the correlation between adenosine triphosphate (ATP) measurements and microbial contamination using a standardized method. Secondarily, analyzing reproducibility of ATP measurements and aerobic colony counts (ACC’s) on the same surface.

**Methods:**

ATP measurements and ACC’s were conducted on 10 pre-defined fomites in a hospital and nursing home setting. Per fomite two ATP measurements and two agar plate measurements were conducted, each measurement was conducted on a 25 cm^2^ surface. Both measurements were compared and analyzed for correlation.

**Results:**

In total 200 paired measurements were conducted, 200 ATP measurements and 200 ACC’s. The mean of all ATP measurements tested on the same surface was calculated, as was for all 200 ACC’s. There was a strong correlation between the mean of two ATP measurements on two different sites on the same fomite (R = 0.800, p < 0.001) as well as between two ACC measurements on the same fomite (R = 0.667, p < 0.001). A much weaker correlation was found between RLU values and ACC’s (R = 0.244, p < 0.001).

**Conclusions:**

Reproducibility of ATP measurements and ACC’s on the same fomite was good. However, the correlation between RLU values and ACC’s on hospital surfaces was much lower. This may be explained by the wide variety of biological material that is measured with ATP, of which the bacterial load is only one of many components. ATP measurement can be used to give a quantifiable outcome for the rating of cleanliness in health care facilities, however the results cannot be translated into the level of microbial contamination.

## Background

Reducing the spread of microorganisms within the healthcare setting is of great importance to limit the amount of nosocomial infections and control antimicrobial resistance. A number of nosocomial pathogens can survive on hospital surfaces for weeks up to months and can easily spread when cleaning is inadequate [1–4]. Measurement of the quality of cleaning can be performed in different ways. Traditionally, environmental swabbing techniques were used to quantify the level of bacterial contamination of surfaces. However, this technique requires several days before results are available and is relatively costly. A novel method for measuring hospital cleanliness is by measuring the amount of adenosine triphosphate (ATP). Originating in the food and beverage industry, measurement of ATP is nowadays frequently used to measure cleanliness of surfaces in hospitals. By measuring ATP, the presence of all kinds of organic material is measured; e.g. microbial contamination and organic contamination (skin flakes, bodily fluids, food scraps, etc.). As the amount of ATP is quantified, ATP measurements give insight into the level of environmental contamination within the healthcare setting. The ATP results are available within seconds which enable immediate feedback.

By enzymatic conversion of ATP into light, the amount of ATP measured is expressed in Relative Light Units (RLU). Therefore, a high RLU readout is indicative for environmental contamination and consequently facilitates the growth and spread of microorganisms.

However, the correlation between the amount of ATP measured and microbial contamination within the healthcare setting is not well documented and various studies report different correlations [5–9]. These studies used different methods to evaluate the correlation between RLU and bacterial growth. The goal of this study was to examine the correlation between RLU readings and aerobic colony count (ACC’s) with a standardized method for measuring, by exactly measuring the same surface area for both measurements (25 cm^2^). Secondarily, we analyzed reproducibility of ATP measurements and ACC’s on the same surface.

## Methods

### Setting

ATP measurements and ACC’s were performed in one hospital and one nursing home. Measurements were conducted on 10 different pre-defined fomites (Table 1). Per fomite two ATP measurements and two ACC measurements were performed. All four measurements were conducted next to each other on the same fomite, without overlap of the measurements. Visual contamination was taken into account and visually contaminated spots were skipped, so that all four measurements were as standardized as possible. The surface area measured with both the ATP-swab and RODAC (Replicate Organism Detection and Counting) agar plate was 25 cm^2^, a plastic template was used to precisely measure 25 cm^2^ and disinfected between measurements. Fomites were sampled at a random point during the day, independent from cleaning rounds. The correlation between ATP measurements on two different sites on the same fomite and the correlation between ACC’s on the same fomite was calculated, assessing reproducibility of both measurements. Furthermore, the correlation between RLU and ACC’s was assessed.

**Table 1:** Overview of measured fomites

Fomite
Table—patient room
Nightstand—patient room
Windowsill team post—outside
Windowsill team post—inside
Computer on wheels—trolley
Bed sheet trolley
Medication trolley
Table—team post
Working table team post × 2

### Measurements

The Clean-Trace NG Luminometer (3M, Zoeterwoude, the Netherlands) was used for the ATP measurements, results were reported in RLU. One trained researcher conducted all ATP measurements. ATP measurements were performed on a flat surface, an area of 25 cm^2^ (5 × 5 cm) was thoroughly swabbed in three directions with an ATP-swab. The manufacturer’s guidelines on conducting ATP measurements were followed.

Measurement of microbial contamination was performed by using tryptone soya agar (TSA) Tween Lecithin 55 mm vp10 agar plates (Biotrading, Mijdrecht, The Netherlands), with a 25 cm^2^ surface. The agar plates were pressed onto the surface for 10 s and afterwards incubated at 35 °C for 48 h, to optimize bacterial and fungal growth. Afterwards the amount of colony forming units (CFU) per agar plate was counted. The maximum amount of countable CFU was fixed on 300.

### Statistical methods

All data were analyzed with Statistical Package for Social Science software (SPSS; IBM Corp., Armonk, New York, US; version 25) and R (R Foundation, New Zealand, R version 3.6.2). The correlation between RLU values and ACC’s was calculated using the Spearman’s rank correlation coefficient.

## Results

In total 400 measurements were conducted on 100 surfaces, consisting of 200 ATP measurements and 200 ACC’s. One hundred paired measurements were conducted in a hospital on different wards, and 100 paired measurements in a nursing home on different wards.

The median RLU value for the total of ATP measurements was 461 with a range from 45 up to 209.520. The median CFU value was 44 with a range from 1 up to 300.

**Fig. 1:**
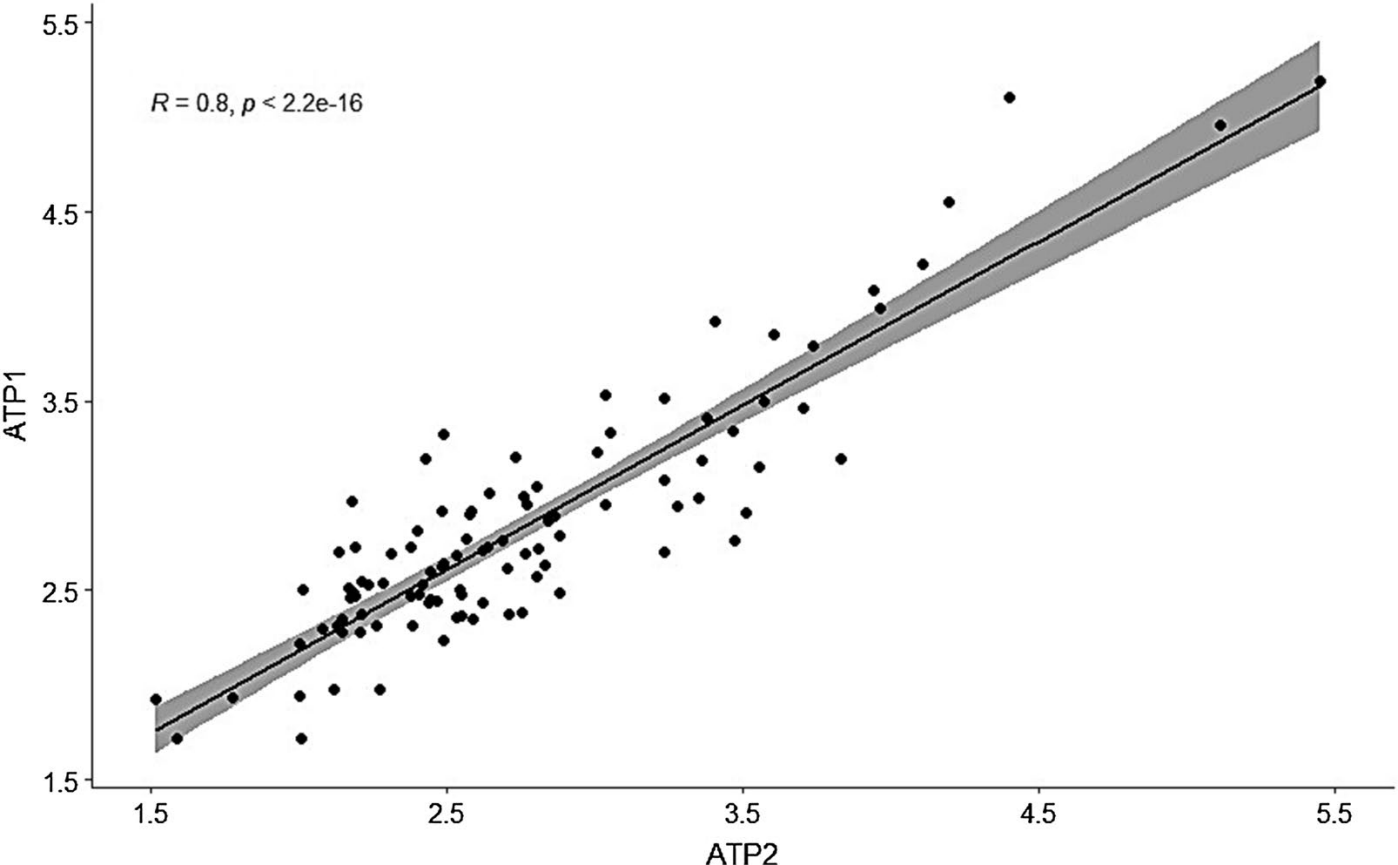
Scatterplot with fit line of ATP measurements (RLU) on the same fomite

**Fig. 2:**
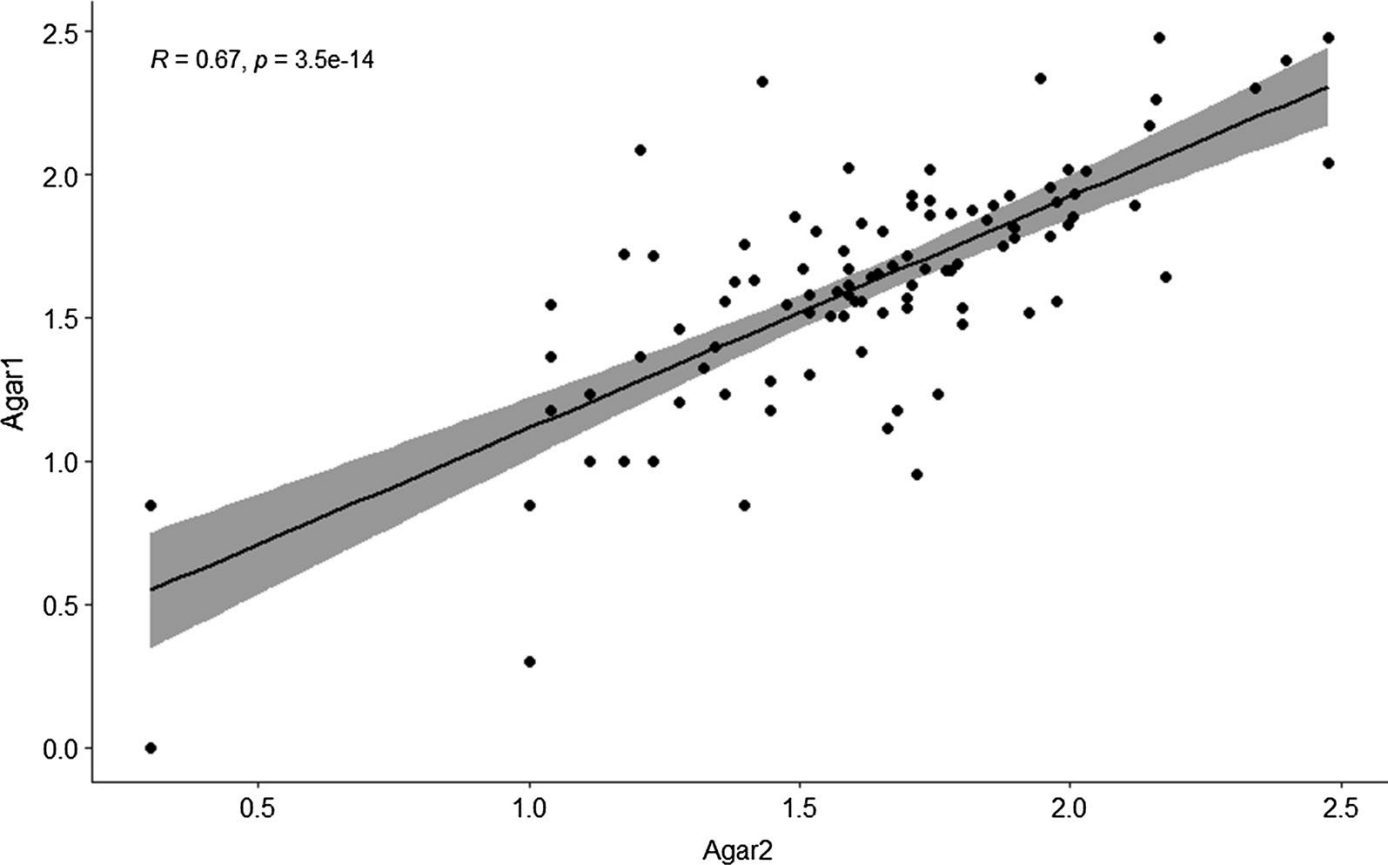
Scatterplot with fit line of microbial measurements (CFU) on the same fomite

There was a strong correlation between two ATP measurements on two different sites on the same fomite (0.800, p < 0.001), Fig. 1. The correlation between two ACC measurements was strong as well (0.667, p < 0.001), Fig. 2.

**Fig. 3:**
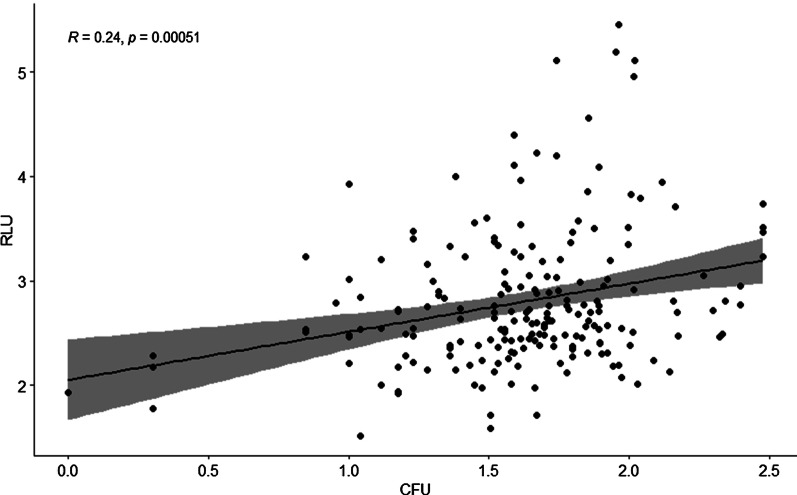
Scatterplot with fit line of logRLU and logCFU

Spearman’s correlation of ATP (RLU) and ACC (CFU) of the 400 measurements showed a weak correlation with a coefficient of 0.244 (p < 0.001). Figure 3 visualizes the correlation between both measurements in a scatterplot, a logarithmic transformation was used for visualizing the data.

## Discussion

To assess the correlation between ATP measurements and ACC’s we conducted measurements on multiple pre-defined identical fomites. Reproducibility was assessed by comparing two measurements on the same fomite.

There was a strong correlation between two ATP measurements on different sites on the same fomite and between two ACC measurements. Furthermore, we found a low correlation between the amount of ATP measured and the amount of microbial contamination.

An explanation for this low correlation could be that other biological materials are measured with ATP measurement. The amount of bacterial/fungal contamination on a surface could be low, while contamination with other biological material (e.g. food scraps, skin flakes, etc.) is higher. ATP measurement is frequently used to quantify surface cleanliness and determining problematic areas. Contamination of surfaces with organic materials could provide a nutritional source for bacteria and other pathogens. By using ATP measurements for analyzing surface contamination, potential nutritional sources for microbes can be traced and disposed. A decrease in microbial growth, contributing to a clean hospital/nursing home environment, can reduce the risk of microbial transmission [10].

Within the hospital and nursing home no outbreaks of multi drug resistant micro-organisms (MDRO’s) were reported. In a setting of an MDRO outbreak the correlation between RLU values and CFU could theoretically be greater. Spread of MDRO’s from a patient within a room could contribute to a higher percentage of measured CFU, thus relatively lowering the percentage of contamination from other sources (e.g. food scraps, skin flakes, etc.). Consequently, the correlation between RLU van CFU could increase.

Another point of discussion is the correlation between the amount of ATP measured and the expressed RLU. Omidbakhsh et al*.* (2014) did further research on this point; they conclude that there is a strong positive correlation between true concentrations of ATP and RLU readings, however this correlation is best when the concentration of ATP is higher. The same conclusion, as described above, is true for the correlation between dilutions of *Staphylococcus aureus* and RLU readings. For the 3M ATP luminometer an R^2^ of 0.9228 was found for the correlation between concentrations of ATP and RLU readings, and an R^2^ of 0.9746 for the correlation between dilutions of *Staphylococcus aureus* and RLU readings [11]. This indicates that there is a good correlation between the amount of ATP/*Staphylococcus aureus* measured and RLU readings. This kind of lab-controlled research gives a better indication of the effect of bacterial load on RLU readings, however is not applicable in daily practice. Contamination with other biological materials can influence RLU readouts, even when bacterial contamination is low. In a sub analysis within this study we did not find a significant difference in the correlation between RLU values and ACC’s with RLU ≥ 1000.

Limitation of ACC are the incubation temperature and selection of growth medium. Various microbes grow with different temperatures and on different culture media [12]. There is a possibility that a certain amount of microbes is missed with the ACC. Thus, giving a lower estimation of microbial contamination than is present on surfaces in reality. By culturing TSA agar plates for 48 h at 35 °C, slow growing microbes and/or those demanding different nutrients could have been missed.

Concluding, the correlation between RLU values and ACC seems to be low. ATP measurement give insight into surface cleanliness and should mainly be used for assessing surface contamination. We found a good correlation between two ATP measurements on the same fomite, indicating that ATP measurement has good reproducibility. Therefore, ATP measurement seems a reliable method for measuring surface contamination.

ATP measurement can be used to give a quantifiable outcome for the rating of cleanliness in health care facilities, however it should not be used to interpret microbial contamination directly.

## Data Availability

Data can be requested by contacting the corresponding author.

## Abbreviations

ATP: Adenosine triphosphate
ACC’s: Aerobic colony counts
RLU: Relative light units
RODAC: Replicate organism detection and counting
TSA: Tryptone soya agar
CFU: Colony forming units
